# Effects of Cardiac Rehabilitation on Sleep Quality in Heart Disease Patients with and without Heart Failure

**DOI:** 10.3390/ijerph192416675

**Published:** 2022-12-12

**Authors:** Fabio Lodi Rizzini, Adela María Gómez-González, Rocío Conejero-Cisneros, María José Romero-Blanco, Almudena Maldonado-Barrionuevo, Pablo Salinas-Sánchez, Manuel Jiménez-Navarro

**Affiliations:** 1 PhD Program in Biomedicine, Translational Research and New Health Technologies, Faculty of Medicine, University of Málaga, 29010 Malaga, Spain; 2 Rehabilitation Department, Virgen de la Victoria University Hospital, 29010 Malaga, Spain; 3 Rehabilitation Department, Costa del Sol Hospital, 29603 Marbella, Spain; 4 Department of Human Anatomy, Legal Medicine and History of Science, Faculty of Medicine, University of Málaga, 29010 Malaga, Spain; 5 Cardiology Department, Virgen de la Victoria University Hospital, IBIMA, CIBERCV, UMA, 29010 Malaga, Spain

**Keywords:** sleep quality, heart failure, cardiac rehabilitation

## Abstract

Insomnia is a modifiable cardiovascular risk factor. Previous studies suggested that attending a cardiac rehabilitation program may improve sleep quality in cardiac patients and pointed out the association between heart failure and poor sleep quality. The primary aim of this study was to evaluate sleep quality in patients attending a Multidisciplinary Cardiac Rehabilitation Program (MRCP), and to compare sleep quality between patients with and without heart failure. A prospective observational study was carried out on a consecutive sample of 240 patients attending an 8-week MRCP; 50 patients (20.8%) were included due to heart failure (NYHA stages I–III) and the rest of them after having undergone any revascularization procedure or valvular surgery. Before and after the completion of the MRCP, the quality of sleep was assessed by the Pittsburgh Sleep Quality Index (PSQI) score. Post-intervention global PSQI scores were statistically significantly lower than those of pre-intervention (*p* = 0.008), but only 60 patients (25%) registered a clinically significant improvement. When comparing patients with heart failure with those without, no differences in sleep quality were found. This suggests that only a small percentage of patients can achieve clinically significant improvements in sleep quality attending conventional MCRP. Suggestions for future research are given.

## 1. Introduction

Sleep disorders are very common in patients who have suffered an episode of acute coronary syndrome [1], with angina pectoris [2] and/or heart failure [3,4,5], as well as in patients who underwent cardiac surgery [6]. Several meta-analyses show that insomnia is a significant risk factor for cardiovascular diseases [7,8,9], therefore interventions addressing sleep quality could in turn also positively affect the risk of developing cardiovascular conditions [10]. More particularly, evidence suggests that not only insomnia but also short and long sleep are significantly associated with obesity, diabetes, hypertension as well as cardiovascular diseases [11].

The correction of cardiovascular risk factors plays a fundamental role in cardiac rehabilitation (CR) programs. However, despite these considerations, the effects of conventional CR programs on sleep quality have received little attention in the current scientific literature; also, in daily clinical practice, this aspect is often neither evaluated nor treated in an individualized form.

Exercise may improve the quality of sleep in healthy adults with sleep problems [12] and may reduce obstructive apnea during sleep [13]. Particularly, physical exercise may improve sleep quality by the means of improving the mood, thereby modulating the activity of sympathetic autonomic nervous system and the release of cytokines, as well as increasing the energy demand which may lead to weight loss and thus improve the occurrence of sleep apnea [14].

Previous studies suggested that attending a CR program may improve sleep quality [15] as well as sleep-associated breathing disorders [16] in cardiac patients. In addition, sleep disorders appear to correlate with depressive symptoms in patients undergoing CR [17], and it has been suggested that a program focusing on cardiac exercise may improve both aspects in these patients [18]. Some works specifically focused on patients with heart failure, reporting similar results [19,20,21,22]. Furthermore, a recent study revealed a moderate significant relationship between sleep quality and physical activity in those patients [23].

However, despite the existing literature highlighting the association between heart failure and poor sleep quality [3,4,5], to our knowledge no studies have been previously conducted in order to evaluate eventual differences in sleep quality between cardiac patients with heart failure and those without.

Therefore, the primary aim of this study was to evaluate baseline values and changes in sleep quality in patients attending a CR program, and to compare differences in sleep quality between patients with and without heart failure. The secondary objectives were to assess baseline values and changes in quality of life, anxiety, depression and functional capacity, as well as to compare eventual differences between the two groups of patients.

## 2. Materials and Methods

### 2.1. Population and Subject Selection

All the patients who joined the CR program at the “Virgen de la Victoria Hospital” in Malaga from March 2018 to May 2019 were considered eligible for this study.

Patients were selected according to the following inclusion criteria: (1) diagnosis of either (any) revascularization (angioplasty or by-pass surgery) within the previous 6 months, hospital admission for heart failure within the previous 6 months, or heart valve surgery (repair or replacement) within the previous 6 months; (2) clinical stability; (3) absence of contraindications to a physical training program.

The following exclusion criteria were also considered: (1) lack of motivation/collaboration from the patient; (2) patient’s refusal to sign the informed consent or participate; (3) comorbidities affecting the musculoskeletal system (e.g., neuromuscular disease); (4) travel time from patient’s residence to the hospital greater than 60 min; (5) severe heart failure (New York Heart Association, NYHA, Class IV: “Unable to carry on any physical activity without discomfort. Symptoms of heart failure at rest. If any physical activity is undertaken, discomfort increases.” [24]).

### 2.2. Intervention

The Multidisciplinary Cardiac Rehabilitation Program (MCRP) implemented was based on programmed physical training, health education and psychological counseling.

The multidisciplinary team in charge of the MCRP included a cardiologist, two physical and rehabilitation medicine (PRM) physicians, a physiotherapist, a nurse and a psychologist. All team members had many years of experience in CR and were trained in basic and advanced cardiopulmonary resuscitation. The PRM specialists were responsible for the design and realization of the MCRP, the assessment of patients eligible for inclusion, the monitoring of patients during CR sessions and the communication with the referring cardiologist.

Before starting the program, patients included underwent risk stratification (low, moderate and high) based on their level of ventricular dysfunction, onset of arrhythmia, signs and symptoms of ischaemia with exercise and METS (metabolic equivalents of task) achieved during the baseline cardiopulmonary exercise test (CPET), based on the guidelines of the Spanish Society of Cardiorespiratory Rehabilitation (SORECAR—Sociedad Española de Rehabilitación Cardiorespiratoria) [25]. Patients then underwent a group-based supervised CR program for 8 weeks, with a frequency of twice weekly sessions for Low Risk patients and thrice weekly sessions for Medium and High Risk ones.

The median wait time to start CR in this study was 17 days [IQR: 10; 21] from risk stratification. During the waiting time the patients were instructed to exercise daily, ideally one hour of walking per day. Exercise heart ratio was determined using the Karvonen formula, applying 60% of the heart rate reserve based on the baseline data from the CPET at the screening visit [26].

Before each training session, the following variables were registered: blood pressure, heart rate, eventual presence of symptoms of chest pain, dyspnoea or discomfort preventing physical exercise.

All the training sessions were supervised by a PRM physician, a physiotherapist and a nurse.

Each training session was structured as follows:

- Warm-up for 10 min, consisting in breathing exercises, joint mobility warm up exercises and stretching;
- Strength training exercises for 20 min, based on short-duration isometric and isotonic exercises. The resistance was set according to the 20 RM (repetition maximum) test; 3 sets of 10 repetitions were used for the upper limbs (biceps, deltoids, triceps and latissimus dorsi) and lower limbs (quadriceps), and the rest interval between sets was 30 s;
- Aerobic training on a cycle ergometer or treadmill, alternating between the two machines from one session to another. Total aerobic training time was 30 min in each session and the target training intensity was calculated according to the Karvonen formula (70–80% for the first and second month of the training regimen, respectively). At the beginning of the MCRP, patients started with two training intervals of 15 min interspersed with a rest period of 5 min. As the patients’ cardiovascular performance improved, they were required to gradually increase the duration of the first training interval until they could maintain the target heart rate continuously for 30 min. For four older patients with HF with reduced ejection fraction, a shorter interval training scheme was initially used consisting of 3 × 10 min training intervals interspersed with 2 × 5 min rest periods. Perceived exertion and breathlessness were constantly monitored using the original Borg Rating of Perceived Exertion (6 to 20 scale), a self-assessment scale of the physical effort made by a patient for a specific workload [27]. We encouraged the participants to maintain a rate of perceived exertion between 13 and 14 points, and exercise was momentarily interrupted for investigations if subjects stated that they had exceeded these thresholds. Target intensity, patients’ Borg Rating of Perceived Exertion and the physiotherapist’s observation of the patient steered the adjustment of the number and length of aerobic training intervals and rest periods during the first training sessions. At the end of the MCRP, all patients were able to maintain the target heart rate continuously for 30 min;
- Relaxation for 10 min, based on breathing exercises and stretching.

At the end of the training session the same variables taken at the beginning were registered, thus verifying the return to the patient’s baseline status.

During the MCRP period, the patients were instructed to continue exercising daily on rest days. As the CR program progressed, they were invited to exercise while maintaining a heart rate equal to that achieved during the hospital training sessions.

Health education was carried out once a week by each professional in the team, to educate and motivate patients and caregivers in controlling the cardiovascular risk factors (CVRF). Groups focusing on nutrition and heart-healthy diet were implemented, as well as individualized advice for obese patients.

Psychotherapy was scheduled once a week through group sessions, with the objective of improving the patient’s quality of life by controlling any emotional changes unrelated to the pathology and acceptance of the untoward pathological event.

### 2.3. Variables Analyzed before the MCRP

- Demographic (age, sex);
- Body mass index (BMI);
- WC: waist circumference;
- Systolic function (LVEF);
- Type of cardiac condition (revascularization, heart failure, heart valve surgery);
- Presence of other CVRF (current or previously smoker, arterial hypertension, dyslipidemia, diabetes mellitus).

### 2.4. Variables Analyzed before and after the MCRP

Sleep quality. The Pittsburg Sleep Quality Index (PSQI) is a self-administered questionnaire assessing the quality of sleep over a one-month interval. It contains a total of 19 items, grouped into 10 questions. The 19 items are combined into 7 areas with a corresponding score: subjective sleep quality, sleep latency, sleep duration, habitual sleep efficiency, sleep disturbance, use of sleep medication and daytime dysfunction. By adding up the scores of the different areas, a global score is obtained that allows discriminating “bad sleepers” (PSQI > 5) from “good sleepers” (PSQI ≤ 5) [28]. In our study we decided to consider a change of 3 or more points to indicate the minimum clinically significant difference, in accordance with Hughes [29].

Quality of life. Quality of life was measured using the Spanish version of the Short Form Health Survey 36 (SF-36) Questionnaire, one of the most widely used instruments to measure health-related quality of life. It consists of 36 questions addressing different aspects of the daily life, grouped in 8 dimensions: physical function, physical role, bodily pain, general health, vitality, social function, emotional role and mental health. The SF-36 also includes a “transition item”, assessing change in general health status compared to the previous year; although not used to calculate any of the dimensions, it still provides useful information on the perceived change in recent health status [30].

Anxiety and depression. The Goldberg Anxiety and Depression Scale (GADS), individually referred to as Goldberg Anxiety Scale (GAS) and Goldberg Depression Scale (GDS), is an 18-item self-report symptom inventory. Each subscale can give a maximum total of 9, with higher scores suggesting greater levels of symptomatology. Generally, anxiety score ≥5 or depression ≥2 shall be deemed as a 50% risk of a clinically important disturbance [31].

Functional capacity. The functional capacity of the patients was expressed in metabolic equivalents of task (METS). A MET represents the amount of oxygen consumed while sitting at rest and is equal to 3.5 mL of oxygen per kilogram per minute. To obtain these values, the Bruce protocol with a cycle ergometer was followed [32], roughly consisting in a maximal exercise test in which the intensity is gradually increased at 3-min intervals. According to literature, the measurement of oxygen consumption during a maximal CPET is considered the gold standard method for assessing cardiovascular functional capacity, since submaximal exercise tests do not accurately predict the maximum rate of oxygen consumption [33].

### 2.5. Data Analysis

Since all 240 patients who started the MCRP finished it, the drop-out rate was 0% and no missing data were registered. Qualitative variables were described by frequency and percentages. The quantitative variables were represented by median, 25th percentile (p25) and 75th percentile (p75). The analyses were performed with RStudio v. 1.0.134. Non-parametric tests were used after verifying that the normality and homoscedasticity criteria necessary to apply the parametric tests were not met. To assess the changes in the outcome measures, median values before and after the treatment, the non-parametric Wilcoxon analysis, was performed. To assess the correlation of the initial and final values of SF-36, GADS, and functional capacity with the PSQI, the Spearman Rho correlation coefficient was used. To assess which characteristics were independently correlated with the PSQI score, a linear regression model adjusted by the least squares method was performed. A comparison of baseline and final data, based on the presence or absence of heart failure, was made performing a bivariate analysis, using the Chi-square or the Mann–Whitney U test. To compare differences between baseline and final values, the Independent Samples Median Test was used.

### 2.6. Data Availability

The data associated with the paper are not publicly available but are available from the corresponding author on reasonable request.

## 3. Results

### 3.1. Subjects Included

A total of 240 patients were included in the study (male: female = 185:55, males = 77.1%), with a median age of 56 years [IQR: 51; 62] and a median BMI of 28.05 [IQR: 26.20; 31.34].

There were 204 of them (85%) who had undergone a revascularization procedure; of these, 174 (85.3%) had been treated with percutaneous transluminal coronary angioplasty (PTCA), 25 (12.3%) with aorto-coronary bypass and 5 (2.4%) with both. A total of 50 patients (20.8%) were included in the program due to heart failure, and 32 patients (13.3%) were included after having undergone surgery for valvular disease. Table 1 shows the demographic and baseline characteristics of the participants.

The 91 patients (37.9%) carried out twice weekly sessions, while 149 (62.1%) managed to complete it thrice, based on risk stratification, as previously explained. As an exception, one patient with heart failure only participated twice a week due to logistical problems in granting his presence to the hospital for more than twice a week, despite his proven motivation in taking part to the program.

### 3.2. Characteristics of the Patients at Baseline and at the End of Follow-Up

**Figure 1:**
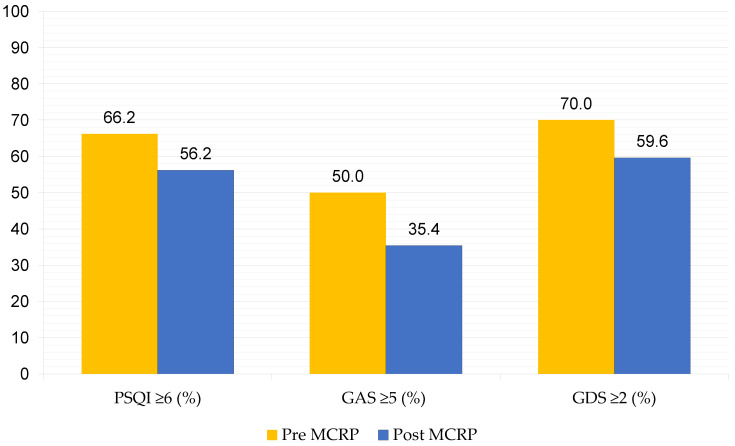
Prevalence (%) of poor sleep quality (PSQI ≥ 6), anxiety (GAS ≥ 5), and depression (GDS ≥ 2) before and after the Multidisciplinary Cardiac Rehabilitation Program. Abbreviations—PSQI: Pittsburgh Sleep Quality Index; GAS: Goldberg Anxiety Scale; GDS: Goldberg Depression Scale; MCRP: Multidisciplinary Cardiac Rehabilitation Program.

A high prevalence of poor sleep quality, anxiety and depressive disorders was observed in our population. Before starting the MCRP, 159 patients (66.2%) had poor sleep quality (PSQI > 5). A total of 120 patients (50%) had anxiety disorder (GAS ≥ 5), while 168 (70.0%) had a depressive disorder (GDS ≥ 2). At the end of the treatment, there were 135 patients with poor sleep quality (56.2%), those with an anxiety disorder were 85 (35.4%), and those with a depressive disorder were 143 (59.6%) (Figure 1).

Table 2 shows the baseline and post MCRP values for sleep quality (PSQI), quality of life (SF-36), anxiety and depression as well as functional capacity.

### 3.3. Changes in Sleep Quality

At the end of the program we observed little improvement in PSQI scale. The median overall score was reduced from 7 to 6 points (*p* = 0.008). In particular, the subscales that registered a change were sleep quality (*p* = 0.023), sleep latency (*p* = 0.023), sleep disturbance (*p* = 0.033) and sleep dysfunction (*p* = 0.023) (Table 2). These changes have been statistically significant, but the improvement could not be considered clinically significant, since less than 3 points [29]. A quantitative analysis of the number of patients who showed a clinically significant improvement was performed: out of the 240, 60 patients registered a clinically significant improvement (25.0%), 163 did not show any clinically significant change (67.9%), while 17 presented a clinically significant worsening (7.1%).

### 3.4. Changes in Quality of Life, Anxiety and Depression Levels

At the end of the program, improvements in quality of life, anxiety and depression levels were found.

The SF-36 subscales that registered a change were physical role (*p* = 0.003), bodily pain (*p* = 0.008), vitality (*p* < 0.001), social function (*p* < 0.001), emotional role (*p* = 0.004), mental health (*p* < 0.001) and health transition (*p* = 0.004) (Table 2).

To quantify the size of the changes in the different subscales, reference can be made to the hypothetical standard changes considered by Wyrwich and colleagues [35]. According to their work, the changes obtained in the physical role (+25 points: moderate effect) and emotional role (+33.33: moderate effect) subscales may be considered clinically significant.

At the end of the program, both the scores in the anxiety (−1.50, *p* < 0.001) and the depression subscale (−1.00, *p* < 0.001) were decreased (Table 2).

### 3.5. Changes in Functional Capacity

At the end of the program, the median functional capacity improved from 7.00 to 8.15 METS (*p* < 0.001) (Table 2).

### 3.6. Relationship between Quality of Sleep, Quality of Life, Anxiety, Depression and Functional Capacity

In our sample, poor sleep quality was associated with quality of life, anxiety and depression. On the contrary, no correlation between PSQI score and functional capacity was found.

Using the Spearman Rho correlation coefficient, we found a weak to moderate negative correlation between PSQI and the eight dimensions of the SF-36 (*p* < 0.001) and a moderate positive correlation between PSQI score and Goldberg scales (*p* < 0.001) (Table 3). 

After applying the multivariate linear regression model, we saw that only SF-36 subscales bodily pain and emotional role, as well as the GAS, independently correlated with the PSQI score (Table 4).

These correlations were those maintained after the conclusion of the program (Table 3 and Table 4).

### 3.7. Differences in Sleep Quality, Quality of Life, Anxiety, Depression, Functional Capacity and Response to Treatment between Patients with and without Heart Failure

Evaluating baseline and final data, we observed differences between patients with and without heart failure in the matter of functional capacity and quality of life. On the contrary, no differences regarding quality of sleep, anxiety or depression were found.

To assess the baseline status of patients, a bivariate analysis of all baseline characteristics was performed between the two groups of patients. Patients with heart failure showed a higher prevalence of moderate to severe systolic dysfunction, lower functional capacity (5.27 vs. 7.10, *p* < 0.001), higher scores in SF-36 subscales emotional role (*p* = 0.043), mental health (*p* = 0.049) and health transition (*p* < 0.001) and lower scores in physical function (*p* = 0.043) and general health (*p* = 0.005). On the contrary, no statistically significant differences were found in sleep quality (*p* = 0.909), anxiety (*p* = 0.092) or depression (*p* = 0.281) (Table 5).

Comparing the characteristics at the end of the program, patients with heart failure showed lower functional capacity (5.68 vs. 9.10, *p* < 0.001), better scores in the SF-36 subscales physical role (*p* = 0.035), emotional role (*p* = 0.002), mental health (*p* = 0.026) and health transition (*p* < 0.001) and lower scores in physical function (*p* = 0.032). (Table 6).

Evaluating the response to treatment between patients with and without heart failure, differences in the improvement in functional capacity could also be observed, greater in patients without heart failure (1.00 vs. 0.25, *p* = 0.005). Patients with heart failure showed a little improvement in SF-36 subscale general health, while patients without heart failure did not (*p* = 0.015), but this difference was not clinically relevant. As for the median changes in sleep quality, remaining SF-36 subscales, plus anxiety and depression, no statistically significant differences could be observed (Table 7).

A comparison of the number of patients who showed a clinically significant improvement was also performed, showing no differences between the two groups (*p* = 0.225).

Since low-risk patients without heart failure underwent twice weekly sessions, while heart failure patients underwent thrice weekly sessions, and in order to assess whether this may have altered the results, a further comparison including only the patients who have undergone thrice weekly sessions was made. Differences in the improvement in functional capacity were confirmed (1.00 vs. 0.30, *p* = 0.017), as well as the absence of differences regarding improvements in sleep quality, quality of life, anxiety and depression (Table A1).

### 3.8. Differences in Response to Treatment between Patients Who Have Undergone Twice Weekly Sessions and Those Who Have Undergone Thrice Weekly Sessions

In our study, low-risk patients underwent twice weekly sessions, while medium and high-risk patients had thrice weekly sessions. The only exception was represented by a patient from the heart failure group who followed the twice a week program due to problems getting to the hospital.

To verify whether this may bias the analysis, the results in median changes obtained for all the variables have been compared, showing no differences when performing treatments twice or thrice a week (*p* > 0.05) (Table A2).

### 3.9. Monitoring for Complications

During the treatment sessions, no major complications of traumatic, orthopedic or cardiovascular nature have been observed that would imply a suspension of the training program. There were 13 patients who experienced an episode of angina pectoris and one an episode of supraventricular tachycardia. These complications were resolved after medical evaluation, without the need of suspending the CR program.

## 4. Discussion

### 4.1. Considerations about Sleep Quality

The prevalence of poor sleep quality in our sample of patients at the beginning of the MCRP was equal to 66.2%, confirming the high frequency of this finding among patients who attend a CR program. Our observations agree with those of other authors. Duarte et al. observed a 76% prevalence of poor sleep quality in a sample of 101 patients included in a CR program [15]. Banack et al., in a sample of 259 patients, detected poor sleep quality in 52% of the subjects with a higher prevalence in subjects with depressive symptoms than in those without (77% versus 31%) [17]. In comparison, in a previous large cross-sectional study, the prevalence of poor sleep quality in the general population was found to be 38% [36].

Our study suggests that heart disease patients who attend an 8-week CR program may improve their quality of sleep, but even if 25% of patients registered a clinically significant improvement in PSQI global score, the overall median change could not reach the minimum clinically significant difference.

These results are consistent with those obtained by other authors in previous studies. Duarte and colleagues observed an improvement in the PSQI of 2.4 points after an intensive CR program (3 h per day, 5 or 6 days a week) carried out in patients admitted for 4 weeks [15]. The fact that the change obtained by these authors was greater than the one obtained in our sample could depend on the higher volumes and frequencies of implementation of the training program; however, despite the authors reporting a 25% improvement in quality of sleep, the magnitude of the mean change could not reach the minimum clinically significant difference. Similarly, Suna and colleagues, in a sample of 106 recently discharged heart failure patients, showed that patients attending a 12-week physical exercise program, carried out twice a week, were significantly more likely to have a clinically meaningful improvement than those receiving only home-based exercise advice; however, neither in this case did the mean change in the PSQI reach the minimum clinically significant difference [21].

The lack of efficacy of standard CR on improving sleep quality has been previously suggested by Risom et al., who carried out a randomized trial on patients recently treated with radiofrequency catheter ablation for atrial fibrillation. These authors observed no difference in sleep quality between the CR and usual care groups, although improvements in sleep quality were noted in both groups [37]. Even if these patients were different from those included in our study, their results suggest that sleep quality may improve over time after hospital discharge in cardiovascular patients; therefore, caution should be used when interpreting the results of studies conducted with no treatment controls.

It is important to point out that in none of the cited studies were there specific interventions carried out that focused on sleep quality. In a recent study, Ghane et al. showed that the addition of a sleep intervention program may lead to clinically significant improvements in sleep quality in CR patients [38], although further studies are needed to confirm their findings.

Although the high prevalence of sleep disorders in patients with heart failure is well documented and studied, to our knowledge no studies have been previously conducted to evaluate eventual differences in sleep quality between cardiac patients with heart failure and those without. About this, in our sample no differences were observed when comparing the two groups. This may be because patients with NYHA stage IV heart failure were not included in our study. In fact, as Suna and colleagues previously pointed out [21], patients with severe heart failure (NIHA IV) have a much higher prevalence of poor sleep quality when compared to class I–III.

### 4.2. Implication for Clinical Practice

Current literature suggests that poor quality of sleep can depend on a combination of predisposing, precipitating and perpetuating factors that should be evaluated in each patient, in order to propose an individualized and effective treatment plan [39]. Therefore, clinical practice guides accordingly suggest that an ideal treatment plan for sleep disorders should include the assessment and treatment of those comorbidities currently considered to be related to poor sleep quality, together with cognitive–behavioral therapy and the optimization of pharmacological therapy [40]. In our opinion, the fact that these factors were not considered and treated individually in our MCRP, and neither by previous authors [15,21], could explain why the median change in PSQI global score could not reach the minimum clinically significant difference.

Considering that the reduction in modifiable CVRF, including poor sleep quality, is one of the main goals pursued by CR programs, we believe these considerations have important implications in clinical practice as should draw clinicians’ attention to the need to screen for this condition upon entry to CR programs, as well as to propose a personalized and multimodal treatment to susceptible patients.

The importance of these factors could also explain the lack of significant differences found when comparing the quality of sleep in patients with and without heart failure. In fact, the results of our study do suggest that, despite patients with heart failure frequently presenting specific symptoms related to poor sleep quality (like cough, orthopnea, paroxysmal nocturnal dyspnea and nocturia) [41]; in patients with NYHA I–III the impact of these factors does not represent the major determinant to explain the high prevalence of sleep disorders, the etiology of which should be considered multifactorial and not specifically related to the pathophysiology of heart failure.

### 4.3. Considerations about Quality of Life, Anxiety, Depression and Functional Capacity

Our findings suggest that cardiac patients who complete a CR program may improve their perceived quality of life, with particular focus on the physical and emotional role dimensions. These results confirm those obtained by Duarte et al., who observed an improvement in all the eight dimensions of the SF-36 [15]. Additionally, our study evidenced a weak to moderate positive correlation between quality of sleep and SF-36 subscales.

At the end of the treatment, patients without heart failure had lower scores in the SF-36 subscales physical role, emotional role, mental health, and health transition. This may be due to the fact that during the period following the revascularization procedure or the valvular surgery, these patients may have experienced more limitations in comparison to the period preceding the acute event; on the contrary, since patients with heart failure have a chronic condition, they would be less likely to experience a deterioration in their state of health compared to the period before the start of treatment, despite having lower levels of physical limitations compared to patients without heart failure.

A high prevalence of anxiety and depressive disorders was observed in our population, together with their association with poor sleep quality. This has been previously described in other studies [17,21]. Abdelbasset and colleagues have shown how aerobic exercise may improve depressive symptoms in patients with heart failure [22], and previous authors pointed out how the implementation of group-based rehabilitation models may improve these aspects by reducing the social isolation, which is characteristic of these patients [42]. As regards our study, an improvement in these very aspects was observed in patients who attended our MCRP.

Patients with heart failure showed a lower baseline value in functional capacity, which did not improve as much as that of patients without heart failure. This result may be due to the fact that patients with heart failure exhibit a decrease in cardiac output during exercise, and as long as cardiac dysfunction associated with heart failure persists, it is not possible to have a normalization of maximum oxygen consumption [43].

### 4.4. Strengths of the Study

Despite the existing literature suggesting that poor sleep quality represents a modifiable risk factor in cardiac patients [7,8,9], there are only a few previously published studies in which the effects of exercise-based CR on sleep quality have been evaluated qualitatively and quantitatively using the PSQI [15,21,37]. We are the first authors who, by critically analyzing the results from literature, pointed out that in none of the studies conducted to date the mean/median changes in the PSQI score reached the minimum clinically significant difference and tried to interpret these findings in the light of current knowledge on sleep disorders.

Furthermore, a strength of the present study was the large sample size which allowed a more precise estimate of the treatment effect and a comparison between patients with heart failure and those without. We demonstrated, for the first time, that heart failure patients (NYHA stages I–III) do not have poorer quality of sleep compared to cardiac patients without it.

### 4.5. Limitations of the Study

The first methodological limitation of this study is the absence of a control group with no treatment. From our point of view, the current evidence [44,45,46,47] of the potential benefits of a CR program would make it ethically unacceptable to refrain from providing such intervention to all of the participants. It would not even be possible to carry out a crossover study, since the patients should start the rehabilitation program as soon as possible.

Secondly, the number of sessions carried out differed among the participants, since low-risk patients had two sessions a week, while medium- to high-risk ones had three. This adjustment, based on individual patient’s risk, allowed us to increase the number of participants to the CR program. On the other hand, to assess whether this choice could bias the analyses, we compared the results obtained for all the variables when performing treatments twice or thrice a week, finding no differences (Table A2). Regarding this issue, it is important to consider that during the MCRP period, the patients were instructed to continue exercising daily on rest days and to log these workouts in a diary. All patients were asked to bring the diary once a week to the hospital in order to be able to record these workouts. Considering 30 min of aerobic exercise for each training session performed in the cardiac rehabilitation unit, and 60 min for each self-training session performed on rest days, we estimated that the total aerobic training volume was very similar for all patients included in the study, while the number of health education and psychological interventions was identical. Specifically, the median number of aerobic training hours achieved during the MCRP period was 38 [IQR: 39; 41.5] for the low-risk patients and 41.5 [IQR: 39; 43.5] for the medium to high-risk ones. Furthermore, we found no correlation between the total volume of aerobic training achieved and median changes in sleep quality (*p* = 0.942).

Thirdly, in our study patients underwent continuous training in which any rest intervals depended on their individual effort tolerance. Current literature suggests that high-intensity aerobic interval training (HIAIT) may be more effective than continuous training for improving cardiorespiratory fitness and quality of life in cardiac patients [48]. However, effects of HIAIT on sleep quality were not evaluated in our study.

### 4.6. Statistical Power of the Study

A preliminary estimate of the appropriate sample size was conducted through a pre-study power analysis.

Taking into account the number of patients finally enrolled in our study, and considering a PSQI mean pre intervention equal to 9.40 and a pre and post PSQI standard deviation equal to 4.1, then based on a previous study [15] we achieved a power >95% at a 5% level of significance to detect a difference after the intervention of at least 3 units.

Similarly, we achieved a power of 0.99 at a level of significance of 5% (two sided) to detect a PSQI median score greater than 2 units in heart failure patients, compared with those without.

## 5. Conclusions

Patients who attend a CR program have a high prevalence of poor sleep quality, which in turn is associated with quality of life, anxiety and depression. Performing an 8-week CR program may improve the quality of sleep and the quality of life in patients with heart disease in the short term, while decreasing their levels of anxiety and depression. However, clinicians should be aware that only a small percentage of patients can achieve clinically significant improvements in sleep quality by attending conventional programs. We consider that further studies will be needed to determine if the addition of personalized and multimodal treatments of sleep disorders could achieve greater improvements in these patients.

Moreover, no statistically significant differences were found in sleep quality and response to treatment when comparing patients with heart failure (NYHA stages I–III) with those without it. Those findings suggest that the high prevalence of poor sleep quality in these patients is not specifically related to the pathophysiology of heart failure. Further studies will be needed to evaluate the differences in sleep quality between cardiac patients with and without heart failure, possibly including patients with NIHA-IV.

## Figures and Tables

**Table 1:** Physical characteristics, type of cardiac condition and Cardiovascular Risk Factors.

General features	
Sex (M/F)	185 (77.1%)/55 (22.9%)
Age [IQR]	56 [51–62]
BMI [IQR]	28.05 [26.20–31.34]
WC [IQR] men	98.1 [95.2–102.8]
WC [IQR] women	90.8 [88.2–96.1]
Systolic Function *	
Normal	117 (48.8%)
Mildly depressed	62 (25.8%)
Moderately depressed	33 (13.8%)
Severely depressed	28 (11.6%)
Type of cardiac condition	
Revascularization	204 (85.0%)
Percutaneous transluminal coronary angioplasty	174 (85.3%)
Bypass	25 (12.3%)
Both	5 (2.4%)
Heart failure	50 (20.8%)
Valve surgery	32 (13.3%)
Cardiovascular risk factors	
Smoker	33 (13.8%)
Smoker before starting the rehabilitation program	115 (47.9%)
Hypertension	167 (69.6%)
Dyslipidemia	172 (71.7%)
Diabetes mellitus	58 (24.2%)
Overweight	115 (47.9%)
Obesity	88 (36.7%)

Abbreviations—M/F: male/female; IQR: interquartile range; BMI: body mass index; WC: waist circumference. Notice: patients undergoing valvuloplasty or heart failure may also have undergone revascularization: in this case they have been counted in each corresponding group. * Systolic Function classification according to the Left Ventricular Ejection Fraction (LVEF), as reported in Lang RM et al., 2015—Normal: LVE F 50% to 70% (midpoint 60%); Mild dysfunction = LVEF 40% to 49% (midpoint 45%); Moderate dysfunction = LVEF 30% to 39% (midpoint 35%); Severe dysfunction = LVEF less than 30% [34].

**Table 2:** Sleep quality, quality of life, anxiety, depression and functional capacity of subjects before and after the Multidisciplinary Cardiac Rehabilitation Program.

		PRE			POST				
	n	Median	p25	p75	Median	p25	p75	Change	*p*
PSQI total	240	7.00	5.00	11.00	6.00	4.00	11.00	−1.00	**0.008**
PSQI Sleep quality	240	1.00	1.00	2.00	1.00	1.00	2.00	0.00	**0.023**
PSQI Latency	240	1.00	0.00	2.00	1.00	0.00	2.00	0.00	**0.023**
PSQI Sleep duration	240	1.00	0.00	2.00	1.00	0.00	2.00	0.00	0.403
PSQI Sleep efficiency	240	0.00	0.00	2.00	0.00	0.00	1.75	0.00	0.070
PSQI Sleep disturbancies	240	1.00	1.00	2.00	1.00	1.00	2.00	0.00	**0.033**
PSQI Sleep drug	240	0.00	0.00	1.00	0.00	0.00	2.00	0.00	0.499
PSQI Sleep dysfunction	240	1.00	0.00	1.00	1.00	0.00	1.00	0.00	**0.023**
SF-36									
SF-36 Physical function	240	67.50	46.25	83.75	70.00	50.00	85.00	+2.50	0.074
SF-36 Physical role	240	0.00	0.00	75.00	25.00	0.00	100.00	+25.00	**0.003**
SF-36 Bodily pain	240	57.50	32.50	80.00	67.50	36.87	90.00	+10.00	**0.008**
SF-36 General health	240	45.00	35.00	55.00	45.00	35.00	60.00	0.00	0.709
SF-36 Vitality	240	45.00	30.00	60.00	50.00	35.00	70.00	+5.00	**<0.001**
SF-36 Social function	240	67.50	42.50	90.00	77.50	55.00	100.00	+10.00	**<0.001**
SF-36 Emotional role	240	66.66	0.00	100.00	100.00	0.00	100.00	+33.34	**0.004**
SF-36 Mental health	240	60.00	44.00	76.00	64.00	48.00	80.00	+4.00	**<0.001**
SF-36 Health transition	240	37.50	25.00	75.00	50.00	25.00	75.00	+12.50	**0.004**
GAS	240	4.50	0.00	7.00	3.00	0.00	6.00	−1.50	**<0.001**
GDS	240	3.00	1.00	5.00	2.00	0.00	4.75	−1.00	**<0.001**
METS	240	7.00	5.30	8.77	8.15	6.60	10.20	+1.15	**<0.001**

Baseline and final values and statistical significance are shown. Abbreviations—p25: 25th percentile; p75: 75th percentile; *p*: *p*-value; PSQI: Pittsburgh Sleep Quality Index; SF-36: 36-Item Short Form Survey; GAS: Goldberg Anxiety Scale; GDS: Goldberg Depression Scale; METS: Metabolic equivalents of task; bold highligted the statistical significant result (*p* < 0.05).

**Table 3:** Correlation coefficients of the initial and final values of SF-36, Goldberg Anxiety Scale, Goldberg Depression Scale and functional capacity with the Pittsburgh Sleep Quality Index.

	PRE		POST	
	Spearman’s Correlation Coefficient	*p*	Spearman’s Correlation Coefficient	*p*
SF-36 Physical function	−0.330	**<0.001**	−0.341	**<0.001**
SF-36 Physical role	−0.348	**<0.001**	−0.388	**<0.001**
SF-36 Bodily pain	−0.453	**<0.001**	−0.473	**<0.001**
SF-36 General health	−0.301	**<0.001**	−0.412	**<0.001**
SF-36 Vitality	−0.403	**<0.001**	−0.406	**<0.001**
SF-36 Social function	−0.429	**<0.001**	−0.427	**<0.001**
SF-36 Emotional role	−0.384	**<0.001**	−0.432	**<0.001**
SF-36 Mental health	−0.522	**<0.001**	−0.569	**<0.001**
SF-36 Health transition	−0.103	0.113	−0.192	**0.003**
GAS	0.529	**<0.001**	0.512	**<0.001**
GDS	0.468	**<0.001**	0.486	**<0.001**
METS	−0.086	0.187	−0.161	0.012

Abbreviations—SF-36: 36-Item Short Form Survey; GAS: Goldberg Anxiety Scale; GDS: Goldberg Depression Scale; METS: Metabolic equivalents of task; *p*: *p*-value; bold highligted the statistical significant result (*p* < 0.05).

**Table 4:** Linear correlation of the initial and final values of the Pittsburgh Sleep Quality Index with respect to the SF-36 and Goldberg scales.

	Baseline			Post MCRP		
	β	95% CI	*p*	β	95% CI	*p*
SF-36 Physical function	−0.002	−0.030/0.020	0.886	−0.009	−0.037/0.020	0.547
SF-36 Physical role	−0.005	−0.020/0.009	0.486	−0.005	−0.020/0.009	0.476
SF-36 Bodily pain	−0.032	−0.054/−0.009	**0.003**	−0.031	−0.052/−0.009	**0.005**
SF-36 General health	0.013	−0.019/0.046	0.416	0.013	−0.020/0.046	0.440
SF-36 Vitality	−0.003	−0.037/0.028	0.878	−0.006	−0.040/0.028	0.742
SF-36 Social function	−0.005	−0.033/0.025	0.731	−0.003	−0.031/0.025	0.830
SF-36 Emotional role	0.004	−0.009/0.016	0.551	0.003	−0.011/0.016	0.693
SF-36 Mental health	−0.048	−0.082/−0.016	**0.006**	−0.050	−0.084/−0.016	**0.005**
SF-36 Health transition				0.017	0.001/0.033	**0.039**
GAS	0.354	0.126/0.575	**0.002**	0.349	0.123/0.575	**0.003**
GDS	0.143	−0.151/0.457	0.339	0.165	−0.128/0.457	0.270
*constant*	10.663		**<0.001**			**<0.001**
*R* ^2^	0.396			0.407		

Abbreviations—MCRP: Multidisciplinary Cardiac Rehabilitation Program; β: beta coefficient; CI: confidence interval; *p*: *p*-value; SF-36: 36-Item Short Form Survey; GAS: Goldberg Anxiety Scale; GDS: Goldberg Depression Scale; *R*^2^: *R*-squared; bold highligted the statistical significant result (*p* < 0.05).

**Table 5:** Comparison of baseline data based on heart failure.

	without HF		with HF		*p*
	N = 190	79.2%	N = 50	20.8%	
	
Male N | %	148	77.9	37	74.0	0.560
Age median | IQR	56.00	10.00	58.00	13.00	0.394
BMI median | IQR	27.80	5.13	28.50	5.55	0.438
WC men median | IQR	97.2	7.2	99.1	8.7	0.427
WC women median | IQR	90.6	6.8	91.4	7.2	0.411
	
PSQI median | IQR	7.00	6.25	7.00	6.25	0.909
Short Form-36 questionnaire					
SF-36 Physical function score median | IQR	70.00	35.00	60.00	31.25	**0.043**
SF-36 Physical role score median | IQR	0.00	75.00	25.00	75.00	0.189
SF-36 Bodily pain score median | IQR	57.50	47.50	67.50	45.00	0.829
SF-36 General health score median | IQR	50.00	25.00	40.00	20.00	**0.005**
SF-36 Vitality score median | IQR	45.00	35.00	45.00	22.50	0.848
SF-36 Social function score median | IQR	67.50	47.50	71.25	47.50	0.604
SF-36 Emotional role score median | IQR	33.33	100.00	100.00	66.67	**0.003**
SF-36 Mental health score median | IQR	56.00	36.00	68.00	29.00	**0.049**
SF-36 Health transition score median | IQR	25.00	51.25	50.00	50.00	**<0.001**
Goldberg scales Median | IQR					
GAS median | IQR	5.00	5.00	3.00	7.00	0.092
GDS median | IQR	3.00	4.00	3.00	3.25	0.281
METS median | IQR	7.10	3.30	5.27	2.04	**<0.001**
Systolic Function *					
Normal function n | %	110	57.9	7	14.0	**<0.001**
Mild dysfunction n | %	56	29.5	6	12.0	
Moderate dysfunction n | %	17	8.9	16	32.0	
Severe dysfunction n | %	7	3.7	21	42.0	

Abbreviations—HF: heart failure; *p*: *p*-value; IQR: interquartile range; BMI: body mass index; WC: waist circumference; PSQI: Pittsburgh Sleep Quality Index; SF-36: Short Form-36 questionnaire; GAS: Goldberg Anxiety Scale; GDS: Goldberg Depression Scale; METS: metabolic equivalents of task; bold highligted the statistical significant result (*p* < 0.05). * Systolic Function classification according to the Left Ventricular Ejection Fraction (LVEF), as reported in Lang RM et al., 2015—Normal: LVEF 50% to 70% (midpoint 60%); Mild dysfunction = LVEF 40% to 49% (midpoint 45%); Moderate dysfunction = LVEF 30% to 39% (midpoint 35%); Severe dysfunction = LVEF less than 30% [34].

**Table 6:** Comparison of final data based on heart failure.

	without HF		with HF		
	N = 190	79.2%	N = 50	20.8%	
	Median	IQR	Median	IQR	*p*
PSQI	6.00	7.00	6.00	4.25	0.288
SF-36 Physical function	70.00	35.00	60.00	26.25	**0.032**
SF-36 Physical role	0.00	75.00	50.00	100.00	**0.035**
SF-36 Bodily pain	67.50	55.00	78.75	55.00	0.072
SF-36 General health	47.50	25.00	45.00	20.00	0.518
SF-36 Vitality	45.00	35.00	52.50	26.25	0.065
SF-36 Social function	72.50	45.00	87.50	45.00	0.311
SF-36 Emotional role	66.66	100.00	100.00	36.30	**0.002**
SF-36 Mental health	64.00	32.00	76.00	37.00	**0.026**
SF-36 Health transition	25.00	50.00	75.00	50.00	**<0.001**
GAS	3.00	6.00	2.50	4.00	0.085
GDS	2.00	5.00	1.50	3.25	0.061
METS	9.10	3.23	5.68	2.43	**<0.001**

Abbreviations—HF: heart failure; IQR: interquartile range; *p*: *p*-value; PSQI: Pittsburgh Sleep Quality Index; SF-36: Short Form-36 questionnaire; GAS: Goldberg Anxiety Scale; GDS: Goldberg Depression Scale; METS: metabolic equivalents of task; bold highligted the statistical significant result (*p* < 0.05).

**Table 7:** Comparison of differences between baseline and final values based on heart failure.

	Without HF		With HF		
	N = 190	79.2%	N = 50	20.8%	
	Median	IQR	Median	IQR	*p*
PSQI	0.00	4.00	−1.00	4.00	0.183
SF-36 Physical function	0.00	15.00	0.00	11.25	0.905
SF-36 Physical role	0.00	25.00	0.00	27.50	0.119
SF-36 Bodily pain	0.00	30.00	10.00	23.13	0.110
SF-36 General health	0.00	20.00	5.00	21.25	**0.015**
SF-36 Vitality	5.00	30.00	7.50	20.00	0.523
SF-36 Social function	0.00	22.50	2.50	32.50	0.721
SF-36 Emotional role	0.00	0.00	0.00	0.00	0.843
SF-36 Mental health	4.00	24.00	6.00	24.00	0.567
SF-36 Health transition	0.00	25.00	0.00	25.00	0.739
GAS	0.00	2.00	0.00	2.25	0.430
GDS	0.00	1.00	0.00	2.00	0.986
METS	1.00	2.60	0.25	1.97	**0.005**

Abbreviations—HF: heart failure; IQR: interquartile range; *p*: *p*-value; PSQI: Pittsburgh Sleep Quality Index; SF-36: Short Form-36 questionnaire; GAS: Goldberg Anxiety Scale; GDS: Goldberg Depression Scale; METS: metabolic equivalents of task; bold highligted the statistical significant result (*p* < 0.05).

## Data Availability

For reasons of confidentiality and privacy of the participants, the data used in this work are not publicly available but can be requested from the corresponding author on reasonable request.

## Appendix A

**Table A1:** Comparison of differences between baseline and final values based on heart failure including only the patients who have undergone thrice weekly sessions.

	without HF		with HF		
	N = 100	67.1%	N = 49	32.9%	
	**Median**	**IQR**	**Median**	**IQR**	***p***
PSQI	0.00	4.00	−1.00	4.00	0.229
SF-36 Physical function	0.00	20.00	0.00	10.00	0.870
SF-36 Physical role	0.00	25.00	0.00	30.00	0.209
SF-36 Bodily pain	0.00	34.37	10.00	23.75	0.168
SF-36 General health	0.00	20.00	5.00	22.50	0.223
SF-36 Vitality	0.00	23.75	10.00	20.00	0.056
SF-36 Social function	0.00	22.50	2.50	32.50	0.387
SF-36 Emotional role	0.00	0.00	0.00	0.00	0.863
SF-36 Mental health	4.00	23.00	8.00	12.00	0.896
SF-36 Health transition	0.00	25.00	0.00	0.00	0.954
GAS	0.00	2.00	0.00	2.50	0.920
GDS	−1.00	2.00	0.00	2.00	0.897
METS	1.00	2.75	0.30	1.89	**0.017**

Abbreviations—HF: heart failure; IQR: interquartile range; *p*: *p*-value; PSQI: Pittsburgh Sleep Quality Index; SF-36: Short Form-36 questionnaire; GAS: Goldberg Anxiety Scale; GDS: Goldberg Depression Scale; METS: metabolic equivalents of task. bold highligted the statistical significant result (*p* < 0.05).

**Table A2:** Comparison of differences between baseline and final values based on weekly treatment frequency.

	2 Sessions/Week		3 Sessions/Week		
	N = 91	37.9%	N = 149	62.1%	
	**Median**	**IQR**	**Median**	**IQR**	***p***
PSQI Median—IQR	0.00	4.00	0.00	4.00	0.856
SF-36 Physical function—IQR	0.00	15.00	0.00	4.00	0.707
SF-36 Physical role—IQR	0.00	25.00	0.00	15.00	0.270
SF-36 Bodily pain—IQR	0.00	32.50	0.00	25.00	0.145
SF-36 General health—IQR	0.00	15.00	0.00	32.00	0.247
SF-36 Vitality—IQR	5.00	30.00	5.00	20.00	0.904
SF-36 Social function—IQR	0.00	22.50	0.00	20.00	0.971
SF-36 Emotional role—IQR	0.00	0.00	0.00	33.00	0.816
SF-36 Mental health—IQR	4.00	24.00	4.00	0.00	0.119
SF-36 Health transition—IQR	0.00	25.00	0.00	24.00	0.856
GAS Median—IQR	0.00	2.00	0.00	2.00	0.351
GDS Median—IQR	0.00	1.00	0.00	2.00	0.987
METS Median—IQR	1.00	2.50	0.74	2.34	0.404

Abbreviations—*p*: *p*-value; PSQI: Pittsburgh Sleep Quality Index; IQR: interquartile range; SF-36: Short Form-36 questionnaire; GAS: Goldberg Anxiety Scale; GDS: Goldberg Depression Scale; METS: metabolic equivalents of task.
